# Monoclonal antibodies and small molecules: on the cutting edge of Alzheimer’s disease therapy

**DOI:** 10.3389/fcell.2026.1766762

**Published:** 2026-04-09

**Authors:** Alberto Ouro, Gabriel A. Ben-Dor, Manuel Debasa-Mouce, Shelly Gulkarov, Joshua De Leon, Mónica Castro-Mosquera, Tomás Sobrino, Anastasia Bougea, Allison B. Reiss

**Affiliations:** 1 NeuroAging Group (NEURAL), Clinical Neurosciences Research Laboratory (LINC), Health Research Institute of Santiago de Compostela (IDIS), Santiago de Compostela, Spain; 2 Centro de Investigación Biomédica en Red en Enfermedades Neurodegenerativas (CIBERNED), Instituto de Salud Carlos III, Madrid, Spain; 3 Department of Foundations of Medicine, NYU Grossman Long Island School of Medicine, Mineola, NY, United States; 4 Department of Medicine, NYU Grossman Long Island School of Medicine, Mineola, NY, United States; 5 First Department of Neurology, Medical School National and Kapodistrian University of Athens, Athens, Greece

**Keywords:** Alzheimer’s disease, antibodies, small molecules, theragnostic, therapy

## Abstract

Alzheimer’s disease (AD) remains a major global health challenge, with prevalence projected to increase dramatically in the coming decades and no effective treatments available. Current therapies offer only symptomatic relief, reinforcing the need for disease-modifying strategies targeting underlying pathogenic mechanisms. Advances in understanding amyloid-β (Aβ) and tau pathology have propelled the development of targeted interventions, particularly monoclonal antibodies (mAbs) and small-molecule therapeutics. Recent anti-Aβ antibodies, such as aducanumab, lecanemab, and donanemab, have demonstrated significant biological activity and reductions in amyloid burden, leading to regulatory approvals that represent important proof-of-concept milestones. However, these therapies face ongoing controversies related to modest clinical efficacy, accessibility, cost, and safety concerns. In parallel, small-molecule development has expanded beyond failed secretase inhibitors toward more refined mechanisms, including tau aggregation inhibition, kinase modulation, mitochondrial stabilization, and anti-inflammatory pathways. These compounds offer advantages in oral administration, blood–brain barrier penetration, and multi-target engagement. Together, mAbs and small molecules represent complementary therapeutic strategies addressing different aspects of AD pathophysiology. Their integration with emerging biomarkers, genetic profiling, and early diagnostic frameworks is driving a transition toward personalized and stage-specific treatment approaches. This review synthesizes current mechanistic insights, clinical evidence, and translational challenges of both modalities, highlighting how their convergence may shape the next-generation of AD therapeutics.

## 1 Introduction

Alzheimer’s disease (AD) is the most common form of dementia, affecting over 50 million individuals worldwide, and its prevalence is expected to triple by 2050 (Javaid et al., 2021). AD is neuropathologically defined by extracellular deposits of amyloid-β (Aβ) peptides forming senile plaques and intracellular accumulation of hyperphosphorylated tau protein into neurofibrillary tangles (NFTs), accompanied by synaptic dysfunction, neuroinflammation, and progressive neuronal loss (Selkoe and Hardy, 2016; Braak and Del Tredici, 2011). Despite extensive research, currently approved therapies—including cholinesterase inhibitors and N-methyl-D-aspartate (NMDA) receptor antagonists—provide only symptomatic benefit and do not modify the underlying disease process (Birks, 2016). The absence of disease-modifying treatments underscores an urgent unmet need for interventions that may alter the course of neurodegeneration.

Over recent decades, growing insights into AD pathogenesis have driven a paradigm shift from symptomatic management toward targeted, mechanism-based therapeutics (Bougea et al., 2025). The amyloid cascade hypothesis, although subject to revision, has served as the conceptual foundation for much of this work (Hardy and Higgins, 1992; Karran et al., 2011). This evolving framework has guided the emergence of innovative therapeutic strategies aimed at preventing or reversing pathological protein aggregation and its downstream neurotoxic effects. Among these, mAbs and small-molecule compounds have gained prominence as complementary modalities at the forefront of AD drug development (Parums, 2024; Cummings J. et al., 2024).

mAbsdesigned to recognize specific Aβ epitopes or aggregated tau species have redefined the therapeutic landscape (Kemppainen et al., 2025). Notable examples include aducanumab, lecanemab, and donanemab, which have demonstrated robust target engagement and reduction of brain amyloid burden in clinical trials (Mintun et al., 2021; van Dyck et al., 2023; Sevigny et al., 2016). The US Food and Drug Administration (FDA) approval of aducanumab in 2021 and lecanemab in 2023 marked historic milestones in the field (Glymour et al., 2022; Daly, 2023). However, these advancements have also provoked debate regarding modest clinical efficacy, high treatment costs, limited accessibility, and safety concerns such as amyloid-related imaging abnormalities (ARIA) (Sperling et al., 2011; Liu and Howard, 2021). Nevertheless, these antibody therapies provide critical proof-of-concept that disease-modifying interventions are biologically achievable in AD.

In parallel, the development of small-molecule therapeutics has continued to evolve, offering several pharmacological advantages: oral bioavailability, blood–brain barrier (BBB) permeability, and lower manufacturing costs (Oliver and Reddy, 2019). Earlier failures of β-secretase (BACE1) and γ-secretase inhibitors prompted the exploration of more nuanced mechanisms, including tau aggregation inhibitors, selective kinase modulators, mitochondrial stabilizers, and anti-inflammatory agents (Yao et al., 2022; Ghosh, 2024). Recent candidates—such as tramiprosate derivatives, tau aggregation inhibitors, and multi-target neuroprotective ligands—are now in advanced stages of development and clinical testing (Congdon and Sigurdsson, 2018; Aisen et al., 2011).

Together, biologic and small-molecule approaches represent convergent strategies targeting complementary aspects of AD pathophysiology. mAbs provide high specificity and robust biomarker engagement, whereas small molecules offer flexibility in targeting multiple disease pathways. The integration of these approaches with advances in fluid and imaging biomarkers, genetic risk stratification (e.g., *APOE ε4* status), and earlier diagnostic tools is shifting AD therapy toward personalized, stage-specific interventions (Zetterberg and Blennow, 2018; Jack et al., 2018).

This narrative review examines the evolving therapeutic landscape of mAbsand small-molecule compounds in AD. We summarize their molecular mechanisms, clinical evidence, and translational challenges, and we discuss how the convergence of biologic and chemical modalities may reshape future directions in AD therapy.

## 2 Basic mechanisms of Alzheimer’s disease

**FIGURE 1:**
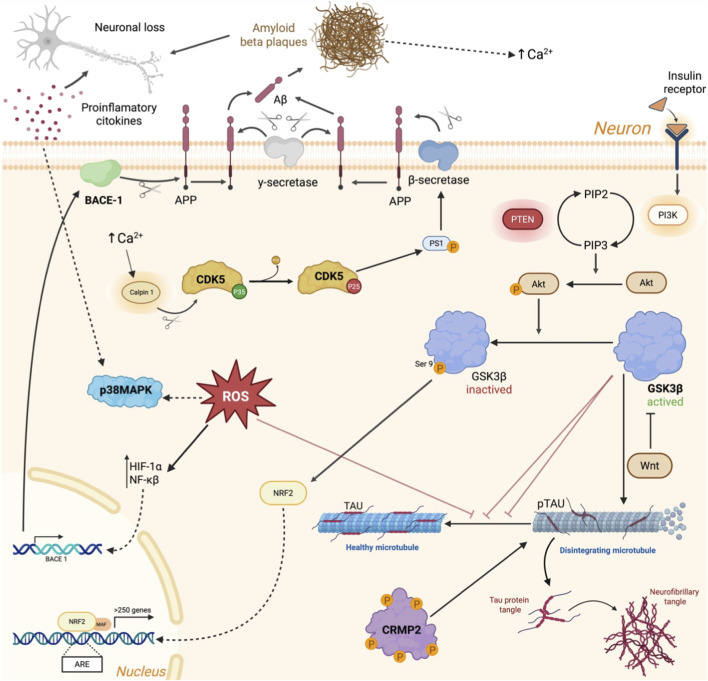
Representation of basic mechanisms involved in Alzheimer’s disease. Beta site amyloid precursor protein cleaving enzyme (BACE); cyclin-dependent kinase 5 (Cdk5); collapsin response mediator protein-2 (CRMP2); glycogen synthase kinase 3 (GSK3); hypoxia-inducible factor 1-alpha (HIF-1α); phosphoinositide 3-kinase (PI3K); protein kinase B (Akt); reactive oxygen species (ROS); nuclear factor erythroid 2-related factor 2 (Nrf2); phosphatidylinositol 4,5-bisphosphate (PIP2); phosphatidylinositol-3,4,5-trisphosphate (PIP3); phosphatase and tensin homolog (PTEN); protein phosphatase 1 (PP1); protein phosphatase 2A (PP2A), soluble APP-beta (sAPPβ); and nuclear factor-kappa B (NF-κB).

In Alzheimer’s disease, two major pathological processes coexist: the extracellular accumulation of β-amyloid (Aβ) peptides and the intracellular aggregation of hyperphosphorylated tau protein leading to neurofibrillary tangles (NFT) formation (Gallardo and Holtzman, 2019). In this regard, different molecular mechanisms have been described as protagonists of the dysregulation of these processes. In this section we will review the most important ones so far. Beta-secretase (BACE) is the protease that triggers the amyloidogenic processing of amyloid precursor protein (APP). This enzymatic action produces amyloid-beta (Aβ) peptides that subsequently aggregate to form amyloid plaques, one of the defining neuropathological features of AD (Vassar et al., 1999). Among the BACE family members, BACE1 is the main isoform implicated in AD (Figure 1). This enzyme is a membrane-anchored aspartic protease predominantly expressed in neuronal cells, whose catalytic region facing the extracellular space performs the first cleavage of APP at the β-site. This step produces soluble APP-beta (sAPPβ) and a membrane-associated C-terminal fragment (CTF-β), which is subsequently processed by γ-secretase to yield Aβ peptides (Yan, 2016). Enhanced BACE1 expression and enzymatic activity have been consistently reported in AD brains, correlating with increased Aβ levels (Holsinger et al., 2002). The excessive accumulation of these peptides contributes to the formation of plaques that trigger downstream pathological processes, including neuroinflammation, tau hyperphosphorylation, synaptic dysfunction, and neuronal degeneration (Vassar et al., 1999). The regulation of BACE1 activity occurs at multiple levels. BACE1 expression is regulated by several factors, such as hypoxia-inducible factor 1-alpha (HIF-1α) and nuclear factor-kappa B (NF-κB), both activated under hypoxia and oxidative stress conditions (Tamagno et al., 2002). Post-translational mechanisms, including phosphorylation, ubiquitination, and glycosylation, further influence in the stability of BACE1, subcellular localization, and enzymatic function.

Glycogen synthase kinase 3 beta (GSK3-β) is a constitutively active, widely expressed proline-directed serine/threonine kinase that participates in diverse cellular processes, including glycogen synthesis, gene transcription, and signal transduction. The enzymatic activity of GSK3 is tightly controlled by multiple phosphorylation-dependent signaling pathways, among which the insulin and Wnt cascades act as its major negative regulators. In the insulin/PI3K/AKT axis, insulin receptor activation leads to PI3K-dependent phosphorylation of AKT, which subsequently phosphorylates GSK3β at Ser9, thereby inhibiting its kinase activity and preventing phosphorylation of downstream substrates such as tau. Conversely, the phosphatase PTEN opposes PI3K signaling, indirectly enhancing GSK3β activity. In the Wnt pathway, GSK3β is sequestered within a multiprotein complex upon Wnt activation, preventing β-catenin degradation and favoring cell survival (Zhao et al., 2024; Albrecht et al., 2021).

Within the AD framework, GSK3β has emerged as a central effector driving multiple pathological features, including tau hyperphosphorylation, amyloid-beta (Aβ) generation, neuroinflammation, and memory decline, linking it to nearly all major hallmarks of AD (Zhao et al., 2024). GSK3β is recognized as one of the primary kinases responsible for tau hyperphosphorylation and subsequent neurofibrillary tangle (NFT) formation (Rankin et al., 2007). Previous studieshave shown that GSK3β can directly phosphorylate tau at multiple residues, promoting its aggregation into filamentous structures resembling those seen in AD. In this regard, around 30 serine or tyrosine residuesin tau sequence are potentially phosphorylated by GSK3 (Hanger et al., 2009). Increased GSK3 expression and activity have been documented both in human AD brains and in animal or cellular models (Lauretti et al., 2020).

Several studies argue that the increase of expression or activity of Ser/Thr kinases such as GSK3β or Cyclin-dependent kinase 5 (Cdk5) decreases the expression of Protein Phosphatase 1 (PP1) and 2A (PP2A), which are phosphatases whose function is to dephosphorylate Tau (Sontag et al., 1996; Gong et al., 1993). In this sense, in AD brains, particularly in the hippocampus, enhanced GSK3β levels correlate with reduced PP2A activity (Vogelsberg-Ragaglia et al., 2001; Pei et al., 1997).

As mentioned, Cyclin-dependent kinase 5 (Cdk5) is a serine/threonine protein kinase belonging to the CDK family. Although it is present in both neuronal and non-neuronal cells, its enzymatic activity is particularly prominent in the nervous system (Shah and Lahiri, 2014). Cdk5 plays a fundamental role in neuronal development, differentiation, and function, as it phosphorylates specific serine and threonine residues on a wide range of substrates involved in these processes (Pao and Tsai, 2021). For its activity, Cdk5 must associate with its regulatory subunit p35, which enables the kinase to perform a broad range of neuronal functions. This Cdk5/p35 complex is essential for neuronal migration and differentiation, synaptic development and plasticity, neurotransmission, and gliogenesis. It also contributes to associative learning, long-term behavioral modulation, retrograde axonal transport, as well as to the formation of cortical layers and the development and proper function of the cerebellum (Quan et al., 2014; Petrik et al., 2013; He et al., 2014; Nishimura et al., 2010). An elevation in intracellular calcium (Ca^2+^) levels can activate calpain, a calcium-dependent protease that cleaves p35 into two fragments, p25 and p10. Under pathological conditions associated with calcium dysregulation, calpain activation promotes this proteolytic event, resulting in p25 generation. The resulting p25/Cdk5 complex retains kinase activity but lacks normal spatial and temporal regulation, leading to a hyperactive form of Cdk5. This aberrant activation triggers multiple neurotoxic mechanisms, including tau hyperphosphorylation, Aβ accumulation, neuronal apoptosis, mitochondrial impairment, cell cycle re-entry, and oxidative stress. Notably, p25 accumulation has been detected in the brains of Alzheimer’s disease (AD) patients, reinforcing the hypothesis that Cdk5 dysregulation driven by p25 formation contributes to AD pathogenesis (Tseng et al., 2002). In addition, Cdk5 can also directly phosphorylate presenilin-1 (PS1) at Thr354, which stabilizes or elevates presenilin protein levels. This modification enhances β-secretase (BACE1) activity, ultimately leading to an increased generation of amyloid-β (Aβ) peptides (Lau et al., 2002). In turn, amyloid-β (Aβ) accumulation elevates intraneuronal calcium levels, leading to calpain activation and subsequent p25 generation, which in turn hyperactivates Cdk5 (Zempel et al., 2010). This establishes a positive feedback loop between Cdk5 and Aβ, perpetuating a cycle that amplifies the pathological processes characteristic of AD.

PP2A constitutes a major family of serine/threonine phosphatases that is abundantly expressed in the brain. Its proper function is essential for neuronal homeostasis, and its dysregulation has been associated with several neurodegenerative diseases. Together with GSK-3β, PP2A acts as a principal regulator of tau phosphorylation. Under physiological conditions, PP2A dephosphorylates tau, thereby preventing its pathological aggregation and maintaining microtubule stability.

Extensive evidence highlights the critical role of PP2A in tau homeostasis, as numerous studies have demonstrated that loss of PP2A activity contributes to the progression of tau pathology in AD (Sontag and Sontag, 2014; Torrent and Ferrer, 2012). Consistently, altered PP2A expression and activity have been detected in postmortem AD brain samples (Baskaran and Velmurugan, 2018). Impairment of PP2A function is strongly associated with tau hyperphosphorylation, amyloidogenesis, and synaptic dysfunction, core pathological features of AD. Moreover, PP2A deregulation affects the activity of several serine/threonine kinases implicated in AD pathophysiology (Sontag and Sontag, 2014). In AD, PP2A/Bα holoenzymes have been shown to interact directly with the microtubule-associated protein tau, emphasizing their key role in tau dephosphorylation and neuronal integrity (Taleski and Sontag, 2018).

Oxidative stress represents another critical factor contributing to PP2A inhibition. The accumulation of reactive oxygen species (ROS) damages proteins, lipids, and nucleic acids, ultimately leading to neuronal dysfunction (Saura and Valero, 2011). In the AD brain, oxidative stress has been shown to suppress PP2A activity while stimulating GSK-3β, resulting in excessive tau phosphorylation and neurotoxicity, a molecular imbalance that characterizes AD pathology (Liu Z. et al., 2015; Qian et al., 2010). Furthermore, ROS-mediated PP2A inhibition enhances GSK3β activation, exacerbating tau hyperphosphorylation and facilitating neurofibrillary tangle formation. Recent evidence also indicates that oxidative stress can promote the dissociation of the PP2A holoenzyme, reducing its enzymatic efficiency and compromising cellular homeostasis (Teleanu et al., 2022; Misrani et al., 2021). Beyond its role in tau regulation, PP2A also influences neuroinflammation through modulation of nuclear factor-kappa B (NF-κB) signaling. In AD, ROS-dependent PP2A inhibition leads to NF-κB activation, which increases the expression of pro-inflammatory cytokines. This inflammatory cascade intensifies oxidative damage and neuronal death, creating a self-perpetuating cycle of neurodegeneration (Clark and Ohlmeyer, 2019).

The PI3K/Akt/GSK3β signaling pathway plays a pivotal role in maintaining tau homeostasis, as AKT-mediated inhibition of GSK3β prevents tau over-phosphorylation (Manning and Toker, 2017). However, neuroinflammatory stimuli, such as C-reactive protein or interleukin-1β (IL-1β), can disturb this regulatory balance, leading to aberrant tau phosphorylation via Akt/GSK3β modulation (Guo et al., 2015). GSK3β also interacts with caspase-3, which cleaves Akt, further increasing tau phosphorylation (Chu J. et al., 2016).

p38 Mitogen-Activated Protein Kinase (p38 MAPK) belongs to the family of MAP kinases that are activated through mitogen- and stress-activated signaling cascades. The PI3K/Akt and MAPK signaling cascades are tightly interconnected, frequently acting in concert to regulate key cellular processes such as growth, survival, and proliferation. Their functional cross-talk plays a crucial role in maintaining cellular homeostasis and is particularly significant in the context of cancer development and therapeutic resistance. p38 MAPK is particularly responsive to cellular stressors, including inflammatory cytokines and ROS. Upon activation, p38 MAPK phosphorylates a broad spectrum of substrates, such as regulatory proteins and transcription factors, thereby influencing numerous cellular processes. In addition, protein kinase R (PKR), which functions upstream of p38 MAPK, can regulate tau synthesis and promote its phosphorylation (Bose et al., 2011). Elevated levels of activated PKR have been detected in both the brain tissue and cerebrospinal fluid (CSF) of AD patients (Mouton-Liger et al., 2012; Paquet et al., 2012). Moreover, genetic inhibition of PKR in the 5xFAD mouse model has been shown to ameliorate cognitive deficits, reduce neuroinflammation and neurodegeneration, and decrease Aβ_1-42_ accumulation (Tible et al., 2019).

In in vivo models, particularly in transgenic mice displaying tau hyperphosphorylation, a strong positive correlation has been observed between p38 MAPK activation and tau aggregation (Kelleher et al., 2007). Consistently, Pei et al. demonstrated elevated p38 MAPK activity in the cortex and hippocampus of both AD patients and mouse models, even at early stages of the disease (Pei et al., 2001). Furthermore, glial overexpression of p38 MAPK has been reported, leading to chronic secretion of pro-inflammatory cytokines by astrocytes and microglia (Bodles and Barger, 2005; Kim et al., 2004).

Importantly, p38 MAPK can directly phosphorylate tau at several residues, enhancing its aggregation propensity and functional impairment (Hanger et al., 2009). These phosphorylation events occur at disease-relevant sites such as Ser202, Thr205, Thr231, and Ser396, all commonly associated with AD pathology (Melone et al., 2018; Jiang et al., 2016). The frequent colocalization of activated p38 MAPK and hyperphosphorylated tau within neurons of AD brains further supports its involvement in tau pathology (Sheng et al., 2001). Additionally, p38 MAPK has been shown to activate GSK-3β and Cdk5 through phosphorylation, thereby amplifying downstream tau phosphorylation cascades.

AMP-activated protein kinase (AMPK), a central regulator of cellular energy metabolism, is also implicated in AD pathogenesis. Reduced AMPK activity is a common feature in AD models, and its upregulation has been shown to counteract tau hyperphosphorylation, whereas GSK3β activators such as wortmannin negate this protective effect, underscoring the reciprocal relationship between AMPK and GSK3β (Zhao et al., 2020).

A key focus in AD research is the interplay between Aβ accumulation and tau pathology, where GSK3β serves as a critical mediator linking these two hallmark processes. Aβ peptides can activate GSK3β and other tau kinases, fostering tau hyperphosphorylation (Vossel et al., 2015; Zhang et al., 2020). Additionally, Aβ-driven neuroinflammation enhances tau pathology through the release of proinflammatory cytokines, notably IL-1β (Terrill-Usery et al., 2014; Gratuze et al., 2018).

Finally, GSK3β contributes to amyloid pathology by modulating APP processing and the activity of BACE1 and γ-secretase, thereby influencing Aβ production (Castro-Alvarez et al., 2015; Chen et al., 2017). Activation of GSK3β promotes amyloid plaque formation, whereas its inhibition diminishes Aβ accumulation in the cortex and hippocampus (Gupta et al., 2022). Interestingly, BACE1 inhibition can paradoxically activate GSK3β, exacerbating tau hyperphosphorylation (Israel et al., 2012).

## 3 Current non-disease modifying AD therapy

Current non-disease modifying therapies for AD primarily focus on managing symptoms, rather than slowing the underlying disease progression. These treatments include cholinesterase inhibitors (like donepezil, galantamine, and rivastigmine) and NMDA receptor antagonists (like memantine) to improve cognitive function, along with neuropsychiatric medications to manage behavioral symptoms like depression and anxiety (Yiannopoulou and Papageorgiou, 2020). According to donepezil’s pharmacodynamic profile, the medication selectively and reversibly inhibits the acetylcholinesterase enzyme, which breaks down acetylcholine in brain cells. AD pathogenesis primarily affects lesion formation in the basal area of the forebrain, which results in a loss of episodic and semantic memories (Haam and Yakel, 2017). The medication’s competitive and uncompetitive inhibition of receptors stops acetylcholine breakdown, which helps AD patients’ memory and cognition. According to clinical evidence from erythrocyte membrane investigations, AChE activity is significantly inhibited at daily dosages of 5–10 mg, with ranges of 63.6%–77.3%.The galantamine works in two ways: first, by interacting with acetylcholinesterase in a reversible manner and raising the amount of ACh in the afflicted area; second, by positively modulating the nicotinic receptors and changing their configuration. Overall, DAD and ADAS improve recovery because of the dual activity of cholinergic neurons in the hippocampus, cortex, and forebrain, which improves memory and behavioral performance (Rockwood et al., 2008). The FDA authorized rivastigmine in 1997, making it the most recently approved acetylcholinesterase inhibitorindicated for mild to moderateAD therapy (Birks, 2016). Rivastigmine is a synthetic derivative based on physostigmine, in contrast to galantamine. It is a carbamate ester that inhibits the acetylcholinesterase enzyme in a non-competitive, long-acting, reversible manner. A synthetic physostigmine derivative called rivastigmine is used to treat mild-to-moderate AD. Rivastigmine works by attaching itself to the cholinesterase enzyme (forms G1, G2, and G4) that is present in the cholinergic neuron’s synapse and hydrolyzing it to stop ACh from degrading (Birks et al., 2015). A dosage of 6–12 mg taken orally has been found to enhance cognition and the mini-mental state evaluation (MMSE) scale. In AD, where there is an overstimulation of glutamate, memantine prevents excessive calcium influx into neurons by blocking NMDA receptors, a process that would otherwise lead to neurotoxicity. Memantine combined with donepezil has been shown to produce superior outcomes for cognition, global assessment, daily activities, and neuropsychiatric symptoms, but lower acceptability than monotherapy and placebo. Combination therapy may also be more cost-effective, because memantine can slow the progression of AD (Guo et al., 2020).

Additionally, non-pharmacological approaches, including nutrition, exercise, and brain stimulation techniques (such as rTMS), are also used to alleviate symptoms and potentially slow disease progression. One of the most promising non-disease modifying therapies for Alzheimer’s disease is cognitive stimulation therapy. This therapy involves engaging patients in a variety of mentally stimulating activities, such as puzzles, memory games, and social interactions. Studies have shown that cognitive stimulation therapy can help improve cognitive function, mood, and quality of life for individuals with AD. By challenging the brain and keeping itactive, cognitive stimulation therapy may help slow the progression of the disease and delay cognitive decline.

Another non-disease modifying therapy for AD is music therapy (Matziorinis and Koelsch, 2022). Music has a unique ability to evoke emotions, memories, and stimulate cognitive function in individuals with AD. Research suggests neural mechanism may involve stimulating brain areas to promote neurogenesis, dopaminergic rewards systems, and the default mode network (DMN) (Slade et al., 2025). Music therapy can help reduce anxiety, depression, and agitation in patients with AD, as well as improve their overall wellbeing (Bleibel et al., 2023). Listening to familiar music or participating in music-making activities can help patients connect with their emotions and memories, providing comfort and a sense of self-expression. Music can improve communication and relationships between patients and their caregivers, which can lead to a reduction in caregiver stress.

Physical exercise is another non-disease modifying therapy that has been shown to have beneficial effects on cognitive function in individuals with AD. Exercise can help improve blood flow to the brain, stimulate the growth of new brain cells, and reduce inflammation and oxidative stress, all of which are important factors in the progression of AD (Pahlavani, 2023). Studies have shown that regular physical activity can help improve memory, attention, and overall cognitive function in individuals with AD, as well as reduce the risk of developing the disease (Yamasaki, 2023; Quigley et al., 2020). This includes slowing cognitive decline, increasing brain volume in memory-related areas, and potentially improving executive function (Mintun et al., 2021). One possible approach is the use of resistance exercise training, given that it has been shown to have cerebroprotective effects associated with AD, such as increasing cortical and hippocampal volume, improving neuroplasticity, and promoting cognitive function throughout the life cycle (Sepúlveda-Lara et al., 2024). However, the exact mechanism is not fully understood.

Nutritional therapy is also being researched as a potential non-disease modifying therapy for AD. Certain nutrients, such as omega-3 fatty acids, antioxidants, and vitamins, have been shown to have cerebroprotective effects and may help reduce inflammation, oxidative stress that contribute to the progression of AD (Xu Lou et al., 2023). However, the evidence for supplements is still limited, and it's important to note that no vitamin or supplement is currently recommended to prevent AD, though some recent studies on multivitamins show preliminary promise for memory and executive function (Kurowska et al., 2023).

Overall, while there is currently no cure for AD, there are several non-disease modifying therapies that offer hope for improving the quality of life for those with the disease. Cognitive stimulation therapy, music therapy, physical exercise, nutritional therapy, and other approaches are helping to address the symptoms and underlying causes of AD, providing new opportunities for patients and their families to effectively manage the challenges of living with this devastating condition. As research continues to advance, it is likely that new and more effective non-disease modifying therapies will continue to emerge, offering hope for a brighter future for those affected by AD.

### 3.1 Degraders as therapeutic tools

As mentioned, the progressive accumulation of aggregates is the molecular hallmark in AD. Notably, these aggregates can be released into the extracellular space and subsequently taken up by other cells, subsequently propagating pathology throughout the brain (Jucker and Walker, 2023).

The ubiquitin–proteasome system (UPS) and the autophagy–lysosomal pathway (ALS) are the main pathways to degrade proteins. Interestingly, aging is associated with a decline in both proteasomal activity and autophagy. This reduction promotes the accumulation of potentially neurotoxic protein aggregates such as Tau and Aβ.

In this regard, different strategies have been developed to selectively degrade therapeutic targets, known as Targeted protein degradation (TPD), that is a fast-increasing field which encompasses different techniques that will be analyzed in this review.

TPD encompasses technologies capable of stimulating or inducing degradation via distinct cellular pathways. This approach has demonstrated its efficacy in the treatment of various cancers (Békés et al., 22022) and neurodegenerative disorders (Kumar and Hassan, 2022), among other inflammatory disease.

The TDP strategy primarily engages two major cellular clearance pathways: the ubiquitin–proteasome system (UPS), which includes PROteolysisTArgeting Chimeras (PROTACs), molecular glues, and hydrophobic tags; as well as the ALP, encompassing Lysosome-targeting chimera (LYTACs), autophagy-targeting chimeras (AUTACs), and Autophagy-Tethering Compound or Autophagosome-Tethering Compound (ATTECs), among others. Substrate specificity is dictated by the cell’s intrinsic protein quality-control mechanisms: UPS-based degraders mainly target misfolded or unfolded polypeptides that can access the proteasomal core, whereas ALP-based degraders are capable of eliminating larger protein aggregates, macromolecular complexes, and even organelles. Consequently, each pathway acts on distinct subsets of proteins within the cell (Fang et al., 2021; Hyun et al., 2021; Hu et al., 2023).

Compared with conventional small-molecule inhibitors, which operate through an occupancy-driven mechanism requiring continuous binding to active sites, often at high concentrations and limited to druggable targets, TPD relies on an event-driven process. By inducing complete degradation of the target protein, TPD permanently abolishes its function. This conceptual and mechanistic shift has considerably expanded the druggable proteome, enabling the selective removal of disease-associated proteins that were previously regarded as undruggable (Hughes et al., 2021; Lin et al., 2021).

Focusing on AD, Chu et al. designed a peptidic PROTAC, termed TH006, that selectively triggers tau protein degradation through the ubiquitin–proteasome system. In an AD mouse model, administration of TH006 markedly enhanced tau clearance within the cortex and hippocampal CA3 region, while simultaneously reducing Aβ-induced neurotoxicity, demonstrating its dual neuroprotective potential (Chu T. T. et al., 2016). Despite their target specificity, these PROTACs exhibited intrinsic physicochemical limitations, notably poor stability and limited membrane permeability, thereby substantially impairing their degradation efficiency (Lu et al., 2018). Furthermore, Wang et al. identified C004019, a small-molecule degrader exhibiting robust tau-degrading activity *in vivo*. This compound demonstrated efficient blood–brain barrier permeability and promoted tau elimination in the brains of 3xTg-AD and hTau transgenic mice. Moreover, C004019 administration led to significant improvements in spatial learning and memory, accompanied by enhanced synaptic function and cognitive performance in the AD mouse model (Wang et al., 2021).

In addition, post-translational modifications (PTMs) are essential regulators of protein stability and function, and their dysregulation has been closely linked to the abnormal protein aggregation characteristic of various neurodegenerative diseases (Zhang et al., 2023). In AD, alterations in tau PTMs play a particularly significant role. Tau hyperphosphorylation impairs its normal interaction with microtubules, leading to cytoskeletal destabilization and promoting pathological self-aggregation. Concurrently, a reduction in O-GlcNAcylation has been observed in AD brains, which further accelerates tau accumulation, microtubule disassembly, and neuronal dysfunction (Wesseling et al., 2020).

To counteract these aberrant modifications, Zheng et al. proposed a novel approach termed a dephosphorylation-targeting chimera (DEPTAC), engineered to recruit tau into proximity with the Bα subunit of protein phosphatase 2A (PP2A), the major tau phosphatase in the central nervous system. Their findings demonstrated that DEPTAC effectively enhanced tau dephosphorylation at several disease-relevant epitopes, resulting in reduced tau burden in both neuronal cultures and transgenic AD mouse models. Remarkably, intracerebroventricular delivery of DEPTAC not only diminished hyperphosphorylated tau but also restored synaptic plasticity, promoted microtubule stabilization, and significantly improved cognitive and learning abilities in AD mice (Zheng et al., 2021).

Despite their therapeutic potential, TACs such as PROTECs and DEPTACs, face substantial limitations in the treatment of neurodegenerative diseases. A primary challenge is their poor BBB permeability, largely attributable to their high molecular weight, polarity and structural complexity. In addition, neurons in the aged or diseased brain often exhibit impaired ubiquitin–proteasome system (UPS) function, rendering proteasome-dependent degradation pathways less effective in these patients. Another critical limitation is the so-called hook effect, whereby PROTACs display a bell-shaped dose–response curve; at high concentrations, simultaneous saturation of the E3 ligase and the target protein prevents productive ternary complex formation, thereby reducing degradation efficiency. Furthermore, many neurodegenerative disorders are characterized by the accumulation of large, misfolded protein aggregates that are more efficiently cleared via autophagy–lysosome pathways rather than by the proteasome. Consequently, conventional proteasome-targeting PROTACs may be suboptimal for eliminating such aggregates. Finally, the widespread expression of commonly exploited E3 ligases, such as CRBN and VHL, throughout peripheral tissues and the central nervous system raises concerns regarding off-target effects and limits the achievement of cell-type-specific protein degradation within the CNS (Mu et al., 2025; Zheng et al., 2025; Cornu et al., 2025).

When considering PROTACs, bispecific antibodies, and other precision protein-targeting modalities, several important limitations warrant attention. The long-term safety of sustained protein depletion remains largely unexplored, raising concerns about adaptive resistance, compensatory pathway activation, and cumulative toxicity (Sakamoto et al., 2003). Although precision protein-targeting strategies are not inherently limited by manufacturability, PROTACs constitute a notable exception. Their bifunctional architecture and catalytic mode of action introduce significant manufacturing and regulatory challenges, as conventional pharmacokinetic and pharmacodynamic (PK/PD) models are insufficient to describe degrader behavior. In addition, difficulties in identifying suitable target ligands—particularly those engaging protein–protein interactions—together with an incomplete understanding of degradation selectivity and off-target effects across biological contexts, complicate rational design and reproducibility. These challenges are further exacerbated by the restricted set of E3 ubiquitin ligases currently exploited, collectively increasing regulatory uncertainty (Gao et al., 2020). Manufacturing and scaling bifunctional small molecules and multispecific antibodies is challenging due to stability, reproducibility, and cost issues. Regulatory pathways are also uncertain, as current frameworks are not fully suited for therapeutics that induce protein degradation rather than inhibit targets (Mullard, 2021; Zhao et al., 2022).

## 4 Monoclonal antibodies therapies in use

First-generation targeting the non-toxic monomeric Aβ, such as solanezumab, bapineuzumab, and crenezumab, failed to demonstrate clinical benefit for AD in clinical trials. Solanezumab targets soluble forms of beta-amyloid, which are believed to be toxic to nerve cells and contribute to the progression of the disease. By binding to soluble beta-amyloid and preventing its harmful effects, solanezumab may help protect nerve cells and slow down the neurodegenerative process in AD. The recent solanezumab incident should serve as an alarm about the unpredictable nature of *post hoc* analysis-based claims. Following the failure of two phase III studies (EXPEDITION-1 and -2), an analysis of the unblinded data revealed that solanezumab was beneficial for mild patients but not for more advanced ones (Doody et al., 2014). A third study, EXPEDITION 3, which was limited to moderate AD dementia, also failed affect cognitive decline (Honig et al., 2018). Both drugs engaged their Aβ targets but neither demonstrated a beneficial effect on cognitive measures compared to controls. Two phase 3 trials (CREAD 1NCT02670083 and CREAD 2NCT03114657) of Crenezumab were discontinued early because an interim analysis showed a low likelihood of success (Ostrowitzki et al., 2022).

The recently developed second-generation anti-amyloid mAbs, such as aducanumab, lecanemab, donanemab, and gantenerumab, target different toxic forms of Aβ aggregates (e.g., oligomers, protofibrils, fibrils, and plaques) (Liu et al., 2023). Aducanumab is a well-known antibody treatment for AD, but its use is controversial due to its approval history, controversial efficacy, and potential side effects. While the FDA approved it in 2021 as the first disease-modifying treatment targeting amyloid plaques, the European Medicines Agency (EMA) recommended against its approval. Aducanumab a human IgG1 anti-Aβ monoclonal antibody selective for Aβ aggregates, which are a hallmark of AD. These plaques are believed to contribute to the development and progression of the disease by disrupting communication between nerve cells and triggering inflammation and cell death. Aducanumab works by binding to beta-amyloid and promoting its clearance from the brain, potentially reducing the buildup of plaques and promoting its removal by the brain’s microglia. So far, two phase III clinical trials EMERGENCT02484547 and ENGAGENCT02477800 showed conflicting results of Aducanumab in mild cognitive impairment and mild dementia due to Alzheimer’s disease (Budd Haeberlein et al., 2022). High-dose of Aducanumab in the EMERGE study showed benefits in the primary outcome (CDR-SB) and in each of the other secondary outcomes (Mini-Mental State Examination [MMSE], Alzheimer’s Disease Assessment Scale—Cognitive—13-item scale [ADAS-Cog-13], and Alzheimer’s Disease Cooperative Study–Activities of Daily Living–for Mild Cognitive Impairment [ADCS-ADL-MCI]), while low-dose Aducanumab did not show benefits compared to placebo. No benefits were seen for low-dose or high-dose ADU in the ENGAGE study in the larger dataset from March 2019. In both the EMERGE and ENGAGE trials, amyloid PET imaging showed dose-related reductions in brain Aβ, indicating target engagement (Knopman et al., 2020). The drug’s continued use and full approval depend on further post-market studies to verify its clinical benefits.

Gantenerumab has a high affinity for aggregated amyloid-beta, including plaques and fibrils, clearing them through microglia (Bateman et al., 2022). Although gantenerumab was not linked to a slower rate of clinical deterioration, it led to in a lower amyloid plaque load than a placebo after 116 weeks in the overall mild-to-moderate AD population. A study in a rare form of inherited AD also failed to show clinical efficacy (Meilandt et al., 2019). Donanemab specifically targets a modified form of beta-amyloid found in the core of amyloid plaques. Clinical trials found that donanemab also significantly reduced levels of tau pathology biomarkers in the blood (p-tau217) and brain, indicating an effect on both pathologies. These antibody therapies are being tested in clinical trials to determine their efficacy and safety in individuals with AD, with the hope of providing new treatment options for this devastating condition.

Lecanemab received traditional approval from the US FDA in July 2023, and has subsequently received approval from the European Medicines Agency (EMA), after a confirmatory trial demonstrated it could slow cognitive decline in early AD by reducing amyloid plaques. Of all second-generation mAbs, lecanemab is the most effective in the immunodepletion of Aβ protofibrils, especially soluble protofibrils and oligomers. This clinical benefit provides evidence supporting the amyloid cascade hypothesis, which posits that the buildup of Aβ is a key driver of the disease (Granzotto and Sensi, 2024). The lecanemab phase 2 study showed rapid Aβ clearance in early symptomatic AD, while the ongoing AHEAD 3–45 trial evaluates lecanemab’s efficacy and safety in cognitively normal subjects with intermediate to elevated Aβ levels. The trial will enroll 400 subjects with intermediate brain amyloid loads and 1000 with elevated Aβ loads. The AHEAD 3-45NCT04468659 trial aims to determine if lecanemab can prevent Aβ accumulation, downstream tau pathology, and cognitive decline in early AD, with a completion date of 2027. The phase III Clarity study of lecanemab demonstrated a significant slowing of decline in clinical (global, cognitive, and functional) outcomes and reduction in brain amyloid in early AD but was associated with adverse events (van Dyck et al., 2023). Table 1 summarizes the main mechanisms of action of FDA approved antibodies therapies for AD.

**TABLE 1:** Summary of pivotal clinical trials for anti-amyloid monoclonal antibodies in Alzheimer’s disease.

mAb (trade name/company)	Pivotal trial (primary endpoint) — exact value (95% CI)	Sample size (randomized/treated)	Key subgroup effects (notable)	ARIA (overall; APOE-stratified where reported)	Notes/clinical interpretation
Lecanemab (Leqembi) — Eisai/Biogen	CLARITY-AD primary (CDR-SB, 18 months): adjusted least-squares mean change 1.21 (lecanemab) vs. 1.66 (placebo); difference = −0.45 (95% CI, −0.67 to −0.23; P < 0.001). (Prespecified primary analysis)	N = 1,795 randomized (898 lecanemab, 897 placebo); safety Core + OLE exposure totals reported in safety paper	Benefit observed across key secondary endpoints (ADAS-Cog14, ADCOMS, ADCS-MCI-ADL). Label/subgroup analyses emphasize benefit in early symptomatic populations; APOE-ε4 status used in regulatory labeling decisions in some regions (EU label restrictions for homozygotes historically debated)	ARIA (Core study): ARIA-E 12.6% (lecanemab) vs. 1.7% (placebo); ARIA-H (microhemorrhage/superficial siderosis) 16.0%–16.9% lecanemab (various reports). APOE: ARIA-E more common in APOE ε4 carriers — Core + OLE: carriers 16.8%; homozygotes 34.5%. Symptomatic ARIA-E ≈3.3% (Core + OLE)	Statistically significant modest slowing of decline (≈20–30% relative slowing by different metrics). ARIA is a class safety issue — more frequent in ε4 carriers and with higher exposures; careful MRI monitoring and patient selection required
Donanemab (Kisunla) — Eli Lilly	TRAILBLAZER-ALZ-2 primary (iADRS, 76 weeks): donanemab showed a statistically significant benefit on the integrated AD Rating Scale (iADRS) at 76 weeks (reported as the primary outcome in JAMA). CDR-SB (secondary/subgroup): in the low/medium tau subgroup LSM CDR-SB at 76 weeks: 1.20 (donanemab) vs. 1.88 (placebo); difference = −0.67 (95% CI, −0.95 to −0.40)	N ≈ 1,736–1,743 randomized across global trial (reported N varies slightly by analysis set; JAMA reported trial sized ∼1,736)	Tau stratification important: efficacy larger in participants with low/medium tau PET burden; subgroup with lower tau had greater slowing. Some secondary/subgroup analyses show larger relative % slowing in low-tau group (hence label guidance often considers tau burden)	ARIA (overall): reported ARIA-E ≈24% in donanemab-treated participants in TRAILBLAZER-ALZ-2 (most radiographically mild/moderate); symptomatic ARIA-E ≈6.1%; serious ARIA (including ICH) rare (∼1.5% serious ARIA-E); some reported treatment-related deaths associated with hemorrhage in small numbers. APOE: ARIA higher in APOE ε4 carriers (detailed stratified rates reported in JAMA and FDA docs). Enhanced titration regimens reduce ARIA incidence in later studies (∼13–14% with modified titration)	Donanemab produced substantial plaque reduction and clinically meaningful slowing in prespecified populations (particularly low/medium tau). ARIA frequency is relatively high — titration strategies under study to improve safety
Aducanumab (Aduhelm) — Biogen/Eisai	EMERGE/ENGAGE (pooled/arm-specific results): trial results were discordant: EMERGE high-dose arm reported a small benefit (example reported EMERGE high-dose difference in CDR-SB ≈ −0.39 in some analyses), while ENGAGE was negative; FDA approval relied on biomarker (amyloid) surrogate and EMERGE high-dose signals — no single consistent prespecified primary trial positive across both	Combined trials ∼3,285 randomized across EMERGE + ENGAGE (high/low dose arms; numbers vary by arm)	Subgroup analyses and dose mattered (high-dose signals drove the positive EMERGE result). APOE ε4 carriers had higher ARIA rates and many trials implemented APOE-stratified monitoring	ARIA: high rates in high-dose arms — ARIA-E up to ∼35% in high-dose pooled reports; ARIA-H also increased (reports vary; microhemorrhage rates increased with dose)	Approval was controversial: biomarker (amyloid) reduction was clear, but clinical benefit small/uncertain and trials discordant — widespread debate about approval basis and clinical meaningfulness. ARIA monitoring and cost/logistics major constraints
Gantenerumab (no trade name) — Roche	GRADUATE I and II (primary: CDR-SB at 116 weeks): pooled/individual trial results: GRADUATE I change from baseline CDR-SB at week 116: 3.35 (gantenerumab) vs. 3.65 (placebo); difference = −0.31 (95% CI, −0.66 to 0.05; P = 0.10) — GRADUATE II: difference −0.19 (95% CI, −0.55 to 0.17); overall not statistically significant for primary endpoints	GRADUATE trials combined N ≈ 2,900+ (each trial ∼1,400–1,500 participants)	Although robust amyloid lowering occurred, clinical endpoints were not significantly improved in prespecified primary analyses; subgroup/sensitivity analyses not convincing for consistent clinical benefit	ARIA: pooled ARIA-E ≈24.9% (gantenerumab), ARIA-H ≈22.9% in study reports; symptomatic ARIA less common (∼5%)	Strong amyloid lowering but phase-3 clinical endpoints failed to meet significance — again underscores plaque reduction ≠ guaranteed clinical benefit. ARIA common
Solanezumab (no trade name) — Eli Lilly	EXPEDITION trials (primary endpoints ADAS-Cog/CDR-SB): multiple large phase 3 trials (EXPEDITION 1–3) did not show significant slowing on primary clinical endpoints (CDR-SB or ADAS-Cog) in mild-to-moderate AD populations (negative trials)	Combined trials >2,000–4,000 participants across programs (EXPEDITION trials each had 1,000+)	Trials suggested no meaningful clinical benefit; later trials attempted preclinical/prodomal populations with no definitive positive result for symptomatic benefit	ARIA: overall low and similar to placebo for solanezumab (targeted soluble monomers—less ARIA compared with plaque-targeting high-clearance agents)	Negative large trials — targeting monomeric soluble Aβ alone was insufficient in symptomatic AD populations tested
Crenezumab (no trade name) — Genentech/AC Immune	CREAD/CREAD2 (phase 3): terminated/negative for primary endpoints — no significant slowing of clinical decline in primary analyses. CREAD study reports show lack of efficacy	CREAD programs N = multiple thousands across trials; CREAD2 stopped early	No convincing subgroup with clear clinical benefit in large trials	ARIA: uncommon/low rates compared with high-clearance plaque antibodies (no major ARIA safety signal comparable to aducanumab/donanemab)	Another large negative program; reinforced that epitope/targeting approach and timing matter
Bapineuzumab (no trade name; Pfizer/Janssen)	Phase 3 trials (Study 301 etc.): failed to demonstrate clinical benefit (primary endpoints negative); some subgroup biomarker signals reported on CSF tau in small subgroups	Large trials (several thousand across dose groups) in mild-to-moderate AD	Post-hoc subgroup analyses suggested possible signals in small subgroups, but not replicated	ARIA: dose-dependent ARIA reported (early trials observed ARIA-E increasing with dose; e.g., up to ∼20% in higher dose groups in some reports)	Early generation fibril-targeting mAb — negative for primary clinical benefit and ARIA emerged as safety concern at higher doses

While antibody therapies show promise as a potential treatment for AD, there are still significant challenges to overcome (Almohmadi et al., 2025). The high cost ranging from 26,500 to over 56,000 per year and require expensive monitoring such as regular MRI or genetic counseling especially for apoE ε4 allele carriers. The main side effect of monoclonal antibody therapies is Amyloid related imaging abnormalities (ARIA) of MRI,which may include brain swelling (ARIA-E) and brain bleeds (ARIA-H) (Doran and Sawyer, 2024). In studies with gantenerumab, donanemab, aducanumab, and lecanemab patients with the apoE ε4 allele have increased association with developing ARIA-E (Wang et al., 2022; Qiao et al., 2023). In addition, the effectiveness of antibody therapies in slowing down the progression of AD may vary depending on the stage of the disease and individual patient characteristics, making it difficult to predict who will benefit most from these treatments.

Bispecific antibodies (bsAbs) are emerging as a promising class of therapeutic agents in the treatment of AD, offering innovative mechanisms to overcome limitations of traditional mAbs (Ma et al., 2021; Yang, 2025). Conventional antibodies targeting amyloid-beta (Aβ) or tau proteins—key pathological hallmarks of AD—have shown modest efficacy due to limited brain penetration, off-target effects, and insufficient clearance of neurotoxic aggregates. Bispecific antibodies, engineered to simultaneously bind two distinct antigens or epitopes, provide a multifaceted approach to disease modification. One design strategy involves combining specificity for Aβ or tau with a receptor that facilitates transcytosis across the blood–brain barrier (BBB), such as the transferrin or insulin receptor (Sehlin et al., 2025a). This “shuttle” mechanism enhances CNS delivery, dramatically increasing therapeutic concentrations within the brain while minimizing systemic exposure. Another promising approach employs bsAbs that simultaneously target different pathological species, such as soluble Aβ oligomers and phosphorylated tau, thereby interrupting the synergistic toxicity and propagation of neurofibrillary tangles (Nam et al., 2025). By engaging both pathologies concurrently, these antibodies could more effectively slow synaptic dysfunction and cognitive decline. Moreover, bsAbs can be engineered to recruit immune effector functions selectively, promoting microglial phagocytosis of aggregated proteins without eliciting excessive neuroinflammation—a common drawback of earlier immunotherapies. Advances in antibody engineering, including Fc optimization and novel linker technologies, have improved the stability, specificity, and safety of bsAbs, making them increasingly viable for clinical development in AD (Ma et al., 2021). Several preclinical studies have demonstrated that bispecific antibodies reduce amyloid and tau burden more efficiently than monospecific counterparts, with improved behavioral and neurophysiological outcomes in animal models of Alzheimer’s and other neurodegenerative diseases (Quint et al., 2021; Sehlin et al., 2025b). Early clinical candidates, such as those coupling anti-Aβ and anti-tau activities, are currently under investigation to assess their safety, pharmacokinetics, and cognitive benefits in human trials (Shastri et al., 2025). While challenges remain—particularly regarding immunogenicity, production complexity, and the precise timing of intervention—bispecific antibodies represent a transformative frontier in Alzheimer’s therapeutics. By integrating enhanced brain delivery and dual-target engagement, these agents hold the potential to shift AD treatment from symptomatic relief toward true disease modification, offering renewed hope for halting or even reversing neurodegenerative progression.

In the context of CNS delivery, these issues are compounded by the limited penetration of conventional IgG antibodies across the BBB, which typically reaches only ∼0.1–0.2% of plasma concentrations (Pardridge, 2005). Receptor-mediated transcytosis strategies targeting the transferrin or insulin receptors can increase brain exposure by 5- to 50-fold; however, high-affinity or bivalent receptor engagement can reduce transcytosis due to endothelial sequestration. Consequently, even optimized BBB-shuttle antibodies generally achieve brain levels of only 0.5%–5% of plasma concentrations, highlighting BBB penetration as a persistent quantitative bottleneck with important implications for dosing and safety (Bien-Ly et al., 2014; Pardridge, 2023).

Future research in AD antibody therapies is focused on developing more targeted and personalized treatments that address the specific molecular and cellular abnormalities underlying the disease. This includes the use of combination therapies that target multiple pathways involved in the progression of AD, as well as the exploration of novel treatment approaches, such as gene therapy and immunotherapy. By addressing the complex mechanisms of AD with innovative antibody therapies, researchers hope to unlock new opportunities for effectively treating and eventually curing this devastating condition.

In conclusion, antibody therapies hold great promise as a potential treatment option for AD, targeting specific proteins and mechanisms involved in the progression of the disease. While challenges exist in terms of efficacy, safety, and accessibility, ongoing research and development efforts are working towards improving the effectiveness and availability of antibody therapies for individuals with AD. The Table 2 summarizes the key different strategies for AD.

**TABLE 2:** Comparative summary of TDP, antibodies and small molecules.

Molecule	Type	Mechanism	Advantages (individual)	Disadvantages (individual)	Advantages	Disadvantages	ref
Degraders	PROTAC	Harnesses the ubiquitin–proteasome system to achieve selective protein degradation, enabling PROTAC molecules to be recycled	• Potential capacity of targeting undruggable proteins and overcome drug resistance • High catalitic activity and selectivity	• Limited oral bioavailability and membrane permeability • Poor ability of aggregated proteins degradation	• Potential capacity of targeting with precision undruggable proteins • Eliminates pathogenic proteins rather than inhibiting them • Effective against aggregates and scaffolding proteins	• Early-stage clinical development • Delivery challenges • Potential safety concerns related to excessive degradation	Hughes et al. (2021); Lin et al. (2021); Zhao et al. (2022); Cai et al. (2024); Schneider et al. (2021)
	DEPTAC	Promotes dephosphorylation of hyperphosphorylated proteins through phosphatase recruitment	• Enables precise modulation of protein functions	• Limited control over the regulation of individual phosphorylation sites			
	LYTAC	Degradation of membrane or extracellular proteins by hijacking the endosomal-lysosomal pathway	• Potential capacity of targeting undruggable proteins • Complete elimination	• Receptor competition • Potential immunogenecity			
	ATTEC and AUTAC	Targets the protein of interest for degradation via the autophagic pathway	• Offers the capacity to clear protein aggregates as well as damaged organelles	• Unclear mechanism of action and potential lysosome-related off-target effects			
Monoclonal antibodies (mAbs)		High-affinity binding to extraxcellular or cell-surface tragets promoting neutralization and/or immune-mediated clerance	• High epitope specificity • Strong biomarker engagement • Proven amyloid clerance in humans		• Reduced to extrecellular and cell surface proteins • Poor BBB penetration; requires high sistemic doses or BBB shuttle strategies • High cost		Ostrowitzki et al. (2022); Budd Haeberlein et al. (2022); Bateman et al. (2022)
Small molecules		Occupancy-driven inhibition (Functional inhibition) or modulation of enzymatic or signaling targets (intracellular or extracellular)	• Oral administration • Good BBB penetration • Weak inmunogenecity • Lower manufacturing costs • Multi-target potential		• Off-target effects • Requires continuous target occupancy • Limited efficacy for aggregation-prone proteins		Manglano-Artuñedo et al. (2024); Yang et al. (2024); Soeda et al. (2019); Jiang et al. (2022); Detka et al. (2024); Zhang et al. (2025b); Mohammed et al. (2025)

### 4.1 The amyloid hypothesis under clinical Scrutiny

The development of mAbstargeting Aβ represents one of the most sustained and expensive therapeutic campaigns in modern medicine, predicated on the assumption that removing Aβ plaques would translate into meaningful clinical benefit for patients with AD (Aisen, 2019; Beveridge et al., 2024). However, while multiple antibodies successfully engaged their molecular target, demonstrating robust plaque clearance on PET imaging, they generally failed to produce clinically meaningful cognitive or functional improvements (Karran and De Strooper, 2022). A 2021 comprehensive meta-analysis examining 18 Phase III trials (solanezumab, bapineuzumab, gantenerumab, crenezumab, and aducanumab) with 21,122 participants quantified this dissociation: amyloid clearance showed very large effect sizes while clinical benefit remained small across most outcome measures (Avgerinos et al., 2021).

This disconnect between biological effect and clinical benefit suggestseither that Aβ plaques are not the primary drivers of neurodegeneration in established AD, or that intervention occurs too late in the disease process, or that the specific epitopes and mechanisms of antibody-mediated clearance matter more than previously appreciated (Haass and Selkoe, 2022; Jin and Noble, 2024; Karran and De Strooper, 2022). The clinical trial failures have forced a critical re-examination of fundamental assumptions about AD pathophysiology and have revealed the complexity of translating molecular biology insights into therapies that improve the lived experience of patients and families.

### 4.2 Solanezumab and bapineuzumab

The first generation of anti-amyloid antibodies to reach Phase III testing, solanezumab and bapineuzumab, established a recurring pattern throughout the field: impressive target engagement without corresponding clinical efficacy. Solanezumab, a humanized IgG1 antibody targeting soluble monomeric Aβ, was designed to shift the equilibrium between soluble and aggregated forms (Doody et al., 2014). In the EXPEDITION NCT00905372and EXPEDITION2NCT00904683 trials (>2,000 mild-to-moderate AD patients) and the subsequent prodromal AD EXPEDITION3 trialNCT01900665,solanezumab failed to meet cognitive endpoints despite target engagement, leading to program termination in 2016 (Lozupone et al., 2024; Honig et al., 2018). Bapineuzumab, an N-terminal anti-Aβ antibody, reduced fibrillar amyloid on PET imaging in a dose-dependent manner but failed to improve clinical outcomes in two large Phase III trials (Salloway et al., 2014). A meta-analysis from Abushouk et al. further supported the lack of benefit and highlighted safety risks (Abushouk et al., 2017).

These trials were notable for revealing a significant safety concern, amyloid-related imaging abnormalities (ARIA), that continues to be a significant focus for the entire treatment class (Qiao et al., 2024). The recognition of ARIA as a distinct class of MRI-detectable safety events led to standardized definitions and monitoring recommendations for ARIA-E (edema/effusion) and ARIA-H (microhemorrhage/superficial siderosis) in anti-amyloid trials (Barakos et al., 2022; Sperling et al., 2011). In subsequent bapineuzumab trials, ARIA-E occurred in approximately 17% of treated participants (versus 0.5% in placebo), with markedly higher rates among APOE4 carriers; ARIA-H was also more common in the treatment group and correlated with APOE4 genotype (Sperling et al., 2012).

The failures of solanezumab and bapineuzumab established that plaque reduction, while necessary, is clearly not sufficient for clinical benefit (Passeri et al., 2022). The ARIA phenomenon revealed that antibody-mediated plaque disruption could trigger vascular complications (Hampel et al., 2023). Third, the lack of efficacy in symptomatic patients raised the question of whether intervention needed to occur earlier in the disease process, during the preclinical or prodromal stages.

### 4.3 Gantenerumab and crenezumab

Learning from the first-wave failures, pharmaceutical developers hypothesized that more aggressive plaque removal might yield clinical benefit (Alam and Sabbagh, 2025). Gantenerumab, a fully human IgG1 antibody with high affinity for aggregated Aβ, was designed for maximize plaque clearance (Bateman et al., 2022; Ostrowitzki et al., 2017). The GRADUATE I NCT03444870and II NCT03443973trials tested high doses in early AD, achieving near-complete plaque clearance in many participants, but in 2022 reports from both trials showed no significant differences from placebo on primary cognitive endpoints (Bateman et al., 2023). Development of gantenerumab has shifted towards a bispecific antibody configuration, trontinemab, which couples gantenerumab to a “Brainshuttle” that allows receptor-mediated delivery to the brain across the blood brain barrier (Grimm et al., 2023).

Crenezumab, an IgG4 antibody designed to minimize ARIA risk through reduced effector function, similarly failed in the randomized, double-blind, placebo-controlled, parallel-group Phase III CREADNCT02670083 and CREAD2NCT03114657 trials despite targeting both oligomeric and fibrillar forms of Aβ (Ostrowitzki et al., 2022). The crenezumab program was terminated in 2019, reinforcing concerns about the fundamental validity of targeting amyloid in symptomatic patients.

These second-generation failures were instructive. Gantenerumab achieved robust plaque clearance without improved clinical outcomes while crenezumab reduced ARIA risk but showed no efficacy. Together, they suggested deeper flaws in the amyloid hypothesis rather than issues of target engagement or antibody design.

### 4.4 The aducanumab controversy

Aducanumab’s trajectory represents a complex and disputed chapter in AD therapeutics. Initially appearing to fail in 2019 when two Phase III trials (EMERGE and ENGAGE) were terminated for futility, the program was resurrected months later based on post-hoc analyses suggesting benefit in the EMERGE trial among patients receiving high doses (Budd Haeberlein et al., 2022; Arnold, 2020). The FDA’s accelerated approval in June 2021—a rigorous regulatory pathway utilizing surrogate endpoints (e.g., amyloid plaque reduction) to address unmet medical needs—was granted despite an advisory committee vote of 0–10–1 against approval. This decision sparked intense debate regarding the interpretation of inconsistent trial results and the reliance on biomarker surrogates for clinical benefit (Alexander et al., 2021). Real-world adoption was minimal, and the sponsor subsequently discontinued marketing of aducanumab in 2024 (Thussu et al., 2024).

### 4.5 Approved therapies: evidence for clinical benefit in selected populations

The FDA approval of lecanemab (January 2023) and donanemab (July 2024) marked the first anti-amyloid antibodies to demonstrate statistically significant clinical benefits in well-designed Phase III trials. Lecanemab, targeting Aβ protofibrils, showed a 27% slowing of cognitive decline on the CDR-SB at 18 months in the Clarity AD trial (Chen et al., 2025; van Dyck et al., 2023). Donanemab, which targets a pyroglutamate-modified form of Aβ in plaques, demonstrated a similar magnitude of effects in the TRAILBLAZER-ALZ 2NCT04437511 trial, with integrated analyses suggesting a 35% slowing on the iADRS at 76 weeks (Rabinovici et al., 2025; Sims et al., 2023). These approvals represented important regulatory milestones, providing the first evidence that amyloid removal can produce measurable cognitive benefits in early AD. However, the magnitude of benefit observed at 18 months, while statistically significant in large trials, raised questions about clinical meaningfulness, as the effect sizes fell below established minimal clinically important difference (MCID) thresholds on most outcome measures (Andrews et al., 2019). The Table 3 summarizes the key mAbs for AD that are either approved, late-stage, or recently discontinued.

**TABLE 3:** Comparative summary of approved and late-stage anti-amyloid mAbs.

Therapy	Target mechanism	Regulatory/Development stage	Clinical efficacy (phase III)	Safety (ARIA risk)	Dosing and administration	Annual cost (est.)
Lecanemab (Leqembi)	Protofibrils (high affinity for soluble protofibrils and oligomers)	FDA Approved (July 2023) and EMA Approved	27% slowing of decline on CDR-SB at 18 months (Difference: -0.45)	ARIA-E: ∼12.6% ARIA-H: ∼17.3% (Risk increases in APOE ε4 carriers).	IV: Biweekly (initiation) Maintenance: Monthly IV or Weekly Subcutaneous (autoinjector approved August 2025)	∼$26,500
Donanemab (Kisunla)	Plaque Core (Pyroglutamate-modified Aβ/N3pG)	FDA Approved (July 2024)	35% slowing on iADRS at 76 weeks; CDR-SB difference −0.67 in low/medium tau populations	ARIA-E: ∼24% ARIA-H: ∼31% (in some reports) (Higher rates than lecanemab; titration used to mitigate).	IV (Titration regimens used to reduce ARIA risk)	∼$32,000
Aducanumab (Aduhelm)	Aggregates (Oligomers, fibrils, and plaques)	Discontinued (Marketing stopped in 2024; originally FDA accelerated approval 2021).Discontinued (Marketing stopped in 2024; originally granted FDA accelerated approval based on surrogate endpoints in 2021)	Conflicting: Positive high-dose arm in EMERGE (−0.39 CDR-SB); negative in ENGAGE trial	ARIA-E: ∼35% (High-dosearm) (Significant safety signal led to monitoring guidelines).	IV (Monthly infusion was standard)	*N/A* (Discontinued)
Gantenerumab	Aggregates (Plaques and fibrils; high affinity)	Failed (Phase III GRADUATE I and II negative; halted)	Negative: No significant slowing of clinical decline despite robust plaque clearance	ARIA-E: ∼24.9% ARIA-H: ∼22.9%	Subcutaneous (Was designed for easier administration)	*N/A*
Solanezumab	Monomers (Soluble Aβ)	Failed (Phase III EXPEDITION 1–3 negative; halted 2016)	Negative: No cognitive benefit observed in mild-to-moderate or prodromal AD.	Low: Rates similar to placebo (due to lack of plaque engagement)	IV	*N/A*

CDR-SB: Clinical Dementia Rating–Sum of Boxes (Primary cognitive endpoint); ARIA-E: Amyloid-Related Imaging Abnormalities–Edema/Effusion.; ARIA-H: Amyloid-Related Imaging Abnormalities–Hemorrhage (microbleeds); IV: intravenous.

### 4.6 Long-term data and biomarker-defined responders

Recent long-term follow-up data on lecanemab suggest that the picture may be more favorable than initial 18-month results indicated, particularly in biomarker-defined subpopulations. At the Alzheimer’s Association International Conference (AAIC) in July 2025, 4-year open-label extension data from Clarity AD were presented, showing expanding benefit over time (https://investors.biogen.com/news-releases/news-release-details/early-alzheimers-patients-continue-benefit-four-years-leqembir). Exploratory comparisons against external natural-history cohorts suggested numerically larger CDR-SB differences at 4 years; however, these findings require confirmation in peer-reviewed reports. These findings suggest that in carefully selected patients with early-stage disease and limited tau pathology, sustained amyloid removal may produce clinically meaningful benefits that accumulate over time.

However, these results must be interpreted with appropriate caution. The data come from an open-label extension comparing treated patients to external natural history cohorts rather than a placebo-controlled continuation, limiting the strength of causal inference. Only 27% of the original trial population (478 of 1,795 enrolled) completed 4 years of treatment, introducing substantial survivor bias (Honig et al., 2024). Those who continued were likely healthier, better responders, and had greater access to resources. The 95% of 18-month completers who chose to continue the open-label extension already represented a selected population. Additionally, identifying low-tau responders requires tau PET imaging, which is not widely available, is expensive, and is typically not covered by insurance, creating a new barrier to implementation. The proportion of real-world early AD patients who would qualify as “low tau” and thus most likely to benefit remains uncertain.

Nevertheless, these findings represent an important conceptual advance: they suggest that short-term trial data may underestimate cumulative disease-modifying effects, and they suggest proof-of-concept that biomarker stratification can identify patient subpopulations with substantially better treatment responses. This potentially points toward a precision medicine approach in which multiple biomarkers (amyloid, tau, neurodegeneration, genetic risk factors) are used not just for diagnosis but for treatment selection and response prediction (Devi, 2023).

### 4.7 The clinical meaningfulness debate: context-dependent benefits

The treatment effects of approved antibodies have generated intense debate about whether statistically significant results constitute clinically meaningful benefits, a controversy with profound implications for treatment recommendations, reimbursement decisions, and research priorities. The 18-month effect sizes from pivotal trials (0.45 points for lecanemab, 0.67 points for donanemab on CDR-SB) fall substantially below established MCID thresholds of 1.0–2.0 points derived from anchor-based analyses linking scale changes to clinician and patient perceptions of meaningful change (Andrews et al., 2019). The regulatory perspective, endorsed by the FDA and reflected across both traditional and accelerated approval pathways, emphasizes that in a progressive, ultimately fatal neurodegenerative disease, any statistically significant and sustained slowing represents meaningful benefit because it prolongs time in higher functional states (Grill and Karlawish, 2021). It is important to note that while accelerated pathways utilize different evidentiary standards—such as relying on validated surrogate endpoints—they are subjected to equally rigorous regulatory assessment (Grill and Karlawish, 2021).

Proponents argue that treatment benefits are cumulative and that longer-term data demonstrate expanding divergence from untreated trajectories (Tysinger et al., 2025). Patient-reported outcomes from Clarity AD (prespecified secondary analyses) could support this view: lecanemab groups showed smaller declines on EQ-5D-5L and QOL-AD, and caregiver burden increased less than with placebo, consistent with preserved daily function (van Dyck et al., 2023). The skeptical interpretation, articulated by many neurologists and methodologists, argues that population-level effect sizes are small and represent changes indiscernible to most patients, families, and physicians in everyday clinical practice (Burke et al., 2023). Critics note that while CDR-SB shows potential slope divergence suggesting disease modification, objective cognitive measures often demonstrate parallel slopes between treatment and placebo groups, raising questions about whether divergence reflects measurement artifacts or genuine disease modification (Knopman et al., 2020).

The clinical meaningfulness debate is further complicated by divergent stakeholder perspectives on what constitutes meaningful benefit. While traditional MCID thresholds derived from anchor-based and distribution-based approaches suggest 1-2 point increases in CDR-SB represent meaningful decline (Andrews et al., 2019), how ‘important’ these changes are regarded may differ substantially among patients, clinicians, healthcare systems, and payers (Cummings, 2025). Patient-reported outcomes from Clarity AD showed 49%–56% preservation of quality-of-life measures, which patients and families may experience as highly meaningful despite falling below established cognitive MCID criteria, while responder analyses focusing on the proportion reaching MCID thresholds remain of particular importance to clinicians (van Dyck et al., 2023). Payers focus primarily on cost-effectiveness ratios and number needed to treat calculations, while family and care partners prioritize quality-of-life impacts and ‘time saved’ in daily functioning (Cummings, 2025; Tarawneh and Pankratz, 2024). These divergent priorities suggest that meaningful benefit may be inherently context-dependent, requiring personalized assessments that incorporate individual values, family circumstances, and stakeholder-specific outcome priorities alongside traditional efficacy metrics.

For patients with very early disease and low tau burden, it remains a possibility that longer longitudinal data in those with sustained treatment may reveal benefits that meet or exceed traditional MCID thresholds. For patients with more advanced disease or higher tau burden, benefits may be statistically detectable but more definitively fall short of practical significance. This heterogeneity may underscore the importance of biomarker-guided patient selection and shared decision-making that considers individual circumstances, preferences, and tolerance for treatment burden (Mittler et al., 2025). The emerging synthesis suggests that clinical meaningfulness may ultimately prove context-dependent, varying with patient characteristics (age, co-morbidities, life-expectancy), disease stage, treatment duration, and individual values.

### 4.8 Real-world implementation challenges

Beyond questions of clinical meaningfulness, approved anti-amyloid antibodies face substantial practical barriers to implementation that limit their population-level impact. The most significant constraint is patient eligibility. Both lecanemab and donanemab require confirmed amyloid positivity (via PET or CSF), early-stage disease (MCI or mild dementia), and the absence of multiple exclusion criteria related to vascular disease, anticoagulation use, and genetic risk factors (Rosen and Jessen, 2025; Defrancesco et al., 2024). Real-world analyses suggest that only 5%–15% of patients with diagnosed AD meet these basic criteria (Tahami Monfared et al., 2022). The identification of low-tau responders through tau PET imaging may further narrow the population most likely to derive benefit; the real-world proportion of early AD patients meeting such criteria remains uncertain and requires confirmation in broader cohorts.

The infrastructure requirements are equally daunting. For lecanemab, patients require an 18-month initiation phase with biweekly intravenous infusions, baseline and periodic MRI monitoring for ARIA (initially before the 5th, 7th, and 14th infusions, then quarterly), and amyloid PET or lumbar puncture for diagnosis (Cummings et al., 2023a). With the field looking toward improving practicality, modeling studies have demonstrated that monthly maintenance dosing can maintain clinical and biomarker benefits after an 18-month initiation phase (Willis et al., 2024), and in January 2025, the FDA approved this maintenance dosing regimen, reducing ongoing treatment burden. Additionally,a subcutaneous autoinjector formulation for weekly maintenance dosing was approved by the FDA on 29 August 2025, and launched in the United States on 6 October 2025. This formulation (lecanemab-irmb subcutaneous injection, branded as Leqembi IQLIK) contains 360 mg/1.8 mL administered via autoinjector over approximately 15 s and is indicated for maintenance dosing following completion of the 18-month intravenous initiation phase (10 mg/kg every 2 weeks). The subcutaneous formulation offers the potential for at-home administration, which may reduce infusion center burden and improve treatment accessibility (Tahami Monfared et al., 2025). However, the 18-month intravenous initiation phase remains required, and subcutaneous administration for treatment initiation is not currently approved.

The surveillance requirements and ARIA burden also contribute real-world application (Bregman et al., 2025). In the lecanemab trials, ARIA-E occurred in 12.6% of treated patients (17.3% in APOE4 carriers), while ARIA-H occurred in 17.3% (31.8% in APOE4 homozygotes) (van Dyck et al., 2023). Although most cases are asymptomatic, serious events including symptomatic hemorrhages and deaths have been reported, particularly in patients on anticoagulation (Wang et al., 2025). Aforementioned, 4-year safety data show that ARIA rates decreased after the initial 12 months and appear to remain stable with continued treatment, providing some reassurance about long-term safety, though the selected population completing 4 years may not be representative of broader real-world patients.

Access disparities compound these challenges. The requirement for specialized imaging and infusion centers concentrates treatment availability in urban academic medical centers, leaving rural and underserved populations with minimal access (Li et al., 2025). Transportation for biweekly (or even monthly) infusions represents a major burden for patients with cognitive impairment and their caregivers. Additionally, racial and ethnic minorities, who face higher AD incidence and worse outcomes, are systematically underrepresented in clinical trials (comprising only 10%–15% of participants despite higher disease burden), raising questions about generalizability of efficacy and safety data to diverse populations (Howell et al., 2016; Zhou et al., 2025). The financial implications remain substantial. With annual list prices at this time exceeding $26,000 for lecanemab and $32,000 for donanemab, plus additional costs for diagnostic imaging, monitoring MRIs, and infusion administration, the total annual cost per patient approaches $80,000–90,000. Cost-effectiveness analyses consistently demonstrate that at current prices, these therapies fall well below accepted willingness-to-pay thresholds in most economic models (Tahami Monfared et al., 2022; Schott and Marshall, 2025).

While this section has largely concentrated on the regulatory landscape in the USA, we recognize the global reach of AD and the regional variability in implementation challenges (Tang et al., 2016; Llibre-Guerra et al., 2023; Dokholyan et al., 2022).

### 4.9 Mechanistic insights from clinical failures

The accumulation of negative and marginally positive trials has generated important insights about AD pathophysiology that extend beyond the original amyloid hypothesis. Perhaps most significantly, they show that even substantial plaque reduction does not reliably yield clinical benefit, as several antibodies achieved over 50% plaque clearance without cognitive improvement (Ackley et al., 2024). This suggests a more complex relationship between amyloid and neurodegeneration than initially theorized, prompting new mechanistic hypotheses (Selkoe and Hardy, 2016).

The timing hypothesis suggests that amyloid deposition occurs years or decades before symptom onset, and by the time patients present with cognitive symptoms, downstream neurodegenerative processes (tau pathology, neuroinflammation, synaptic loss, neuronal death) have become self-sustaining and independent of amyloid (Insel et al., 2021; Jack et al., 2018). This model is supported by biomarker studies showing that amyloid accumulation plateaus early, while tau and neurodegeneration progress linearly throughout the disease course (Jack et al., 2009; Collij et al., 2021). Better response to lecanemab in patients with low tau burden supports this model and could suggest that intervention before extensive tau spread is critical for meaningful benefit.

Another explanation focuses on the specific forms of amyloid targeted. Mounting evidence suggests that soluble oligomeric species, rather than insoluble plaques, may be the primary neurotoxic forms of Aβ (Tolar et al., 2021; Benilova et al., 2012). Most approved antibodies primarily target fibrillar plaque, and the process of antibody-mediated plaque disaggregation may transiently increase oligomeric species, potentially explaining the lack of clinical benefit despite plaque clearance. Lecanemab’s targeting of protofibrils (an intermediate between oligomers and fibrils) may explain its superiority over earlier antibodies, though this remains somewhat speculative.

A more radical challenge to the amyloid hypothesis comes from reconceptualization of Aβ’s role in AD pathogenesis. The Aβ42 hypothesis, articulated by Espay and colleagues, proposes that AD may result not from overproduction or accumulation of Aβ, but from loss of normal Aβ42 function, with plaques representing a failed compensatory response rather than a cause of disease (Abanto et al., 2024; Espay et al., 2019). In this model, removing plaques would have minimal benefit or could even be harmful, a prediction consistent with many clinical trial results. While controversial, this hypothesis highlights how the pattern of trial failures has prompted fundamental reconsideration of disease mechanisms.

The ARIA phenomenon offers mechanistic insights. The higher rates of ARIA-E in APOE4 carriers and the association with rapid plaque clearance suggest that antibody-mediated disruption of vascular amyloid deposits can compromise the blood-brain barrier (Foley and Wilcock, 2024; Greenberg et al., 2020). This underscores cerebral amyloid angiopathy as a distinct pathological process that may warrant different treatments sincethe presence of vascular amyloid may actually contraindicate aggressive plaque-clearing strategies (Foley and Wilcock, 2024).

### 4.10 Future directions: precision medicine and combination approaches

The experience with anti-amyloid antibodies, both failures and qualified successes, points toward several critical directions for future AD therapeutics. The oncology field provides an instructive parallel: cancer treatment evolved from single-agent chemotherapy to multi-target combination regimens that simultaneously address different aspects of tumor biology (Jicha et al., 2024). A similar paradigm shift may be necessary in AD, where multiple pathological processes (amyloid, tau, neuroinflammation, metabolic dysfunction, vascular disease) contribute to neurodegeneration (Cummings et al., 2023b).

Early combination trials are testing anti-amyloid antibodies with anti-tau antibodies, BACE inhibitors, or anti-inflammatory agents (Angioni et al., 2025; Cummings J. L. et al., 2024). The hypothesis is that simultaneously targeting amyloid and tau, the two pathological hallmarks of AD, may produce synergistic benefits greater than either approach alone (Hossain and Hussain, 2025). The potential identification of low-tau responders to lecanemab suggests that combination approaches might be particularly valuable in patients with more advanced tau pathology, where anti-amyloid monotherapy appears insufficient. Precision medicine approaches that match therapies to individual biomarker profiles (amyloid-predominant vs. tau-predominant vs. mixed pathology) may improve treatment responses and allow more personalized risk-benefit assessments (Young et al., 2022).

The experience with anti-amyloid antibodies has also reinvigorated interest in prevention trials in preclinical populations. If the timing hypothesis is correct, that amyloid removal is most effective before extensive downstream neurodegeneration is established, then intervention in cognitively normal individuals with biomarker evidence of amyloidosis may be necessary to achieve disease prevention rather than merely slowing progression. The A4 and AHEAD studies are testing this hypothesis, though results are years away and ethical concerns about treating asymptomatic individuals with agents carrying significant risks remain unresolved (Schott, 2025; Sperling et al., 2023).

The history of anti-amyloid antibody development represents both genuine scientific progress and a cautionary tale about the complexity of neurodegenerative disease. The lessons learned extend beyond specific trial results to fundamental insights about AD pathophysiology. Looking forward, the field appears to be moving toward more sophisticated approaches such as combination therapies, biomarker profiles including both amyloid and tau, earlier intervention, and practical improvements in drug delivery.

### 4.11 Molecules: on the cutting edge of Alzheimer’s disease therapy

Small-molecule drugs have been developed to target specific mechanisms in AD pathogenesis (Yao et al., 2022). Some notable examples include cholinergic drugs, NMDA inhibitors, anti-amyloid agents, many targeting BACE1, and tau aggregation inhibitors.

Cholinergic inhibitors are pertinent small molecules for discussion since their use is widespread and common (Chen et al., 2022). Small molecule-based acetylcholinesterase (AChE) inhibitors were developed based on the cholinergic hypothesis which postulates that loss of cholinergic neurons underlies memory deficits and cognitive decline in AD (Hampel et al., 2018). Acetylcholine plays an important role in cognitive functions related to learning and memory and in the AD brain, degeneration of cholinergic neurons in the nucleus basalis of Meynert has been shown to occur (Liu A. K. L. et al., 2015; Mieling et al., 2023). Cholinesterase inhibitors prevent the hydrolysis of acetylcholine, thereby increasing the level of acetylcholine and prolonging its activity at the synapse (Pozzi et al., 2022; Marucci et al., 2020). While not a cure, they can slow cognitive decline, manage symptoms and decrease mortality (Zuin et al., 2022). The FDA has approved several drugs in this class, including donepezil, rivastigmine, and galantamine, and most recently benzgalantamine, a prodrug form of galantamine with less gastrointestinal side effects (Bakker et al., 2020; Boroje et al., 2025; Andrade et al., 2025). Tacrine, the first clinically approved cholinesterase inhibitor, has been withdrawn due to high hepatotoxicity (Bubley et al., 2023).

Memantine is a small molecule drug that acts as a non-competitive moderate affinity inhibitor of N-methyl-d-aspartate (NMDA) receptors (Boroje et al., 2025; Karimi Tari et al., 2024). Memantine is believed to exert its effects by reducing excessive glutamate release, thus preventing overstimulation of neurons and neuronal apoptosis that may result from excitotoxicity (Cimino and Feligioni, 2024). It is currently approved for the treatment of moderate and severe AD and improves symptoms such as impaired memory and cognitive decline (Tang et al., 2023). Donepezil can be used in combination with memantine in later stages of AD (Conway, 2020; Yaghmaei et al., 2024).

Much effort has been invested into developing AD drugs based on pathological mechanisms related to amyloid and tau. Many have entered clinical trials, and while some can slow down disease progression, they are far from curative.

Despite doubts about the amyloid hypothesis, diverting APP from the amyloidogenic pathway by reducing APP cleavage at the β-site by the BACE1 enzyme has been thoroughly explored as an AD treatment (Iram et al., 2024; Zhang B. et al., 2025). BACE1 inhibition was considered an appealing mechanism of AD treatment because there are oral drugs available at low cost that can accomplish the reduction of Aβ, but clinical trials have been disappointing (Bazzari and Bazzari, 2022). Verubecestat, the first small-molecular BACE1 inhibitor to proceed through to phase 3 trials, failed to show functional benefit with no improvement in cognition in mild-to-moderate AD patients (Egan et al., 2018). It also conferred no benefit in prodromal AD (Egan et al., 2018). In addition, risks associated with verubecestat included increased falls and injuries, suicidal ideations, weight loss, sleep disturbances, and rashes (Egan et al., 2019). Study of the drug has been discontinued (Tang et al., 2023).

Elenbecestat (CNP-520), a BACE inhibitor with oral bioavailability and selectivity for BACE1 over BACE2, was tested in two phase III studies, but both were discontinued due to safety concerns and efficacy issues (Ghosh, 2024). With few exceptions, BACE1 inhibitors are no longer being actively pursued as a therapeutic approach for AD because of the mounting setbacks in clinical trials (Doggrell, 2019).

Given the setbacks observed with BACE1 inhibitors, small molecule drugs directed at Aβ itself have gained some attention. An example is valiltramiprosate (ALZ-801), an oral, small-molecule inhibitor of Aβ oligomer formation being studied in a Phase 3 trial in persons homozygous for ApoE4 with early AD (Hey et al., 2025; Abushakra et al., 2024). This prodrug inhibits Aβ formation through both nucleus formation and fibril elongation (Campagna et al., 2025). Pre-clinical cell-culture studies such as Maramatsu et al. provide mechanistic support for the ability of valiltramiprosate to inhibit both the nucleation phase and elongation phase of Aβ aggregation, with reduction of the cytotoxicity associated with smaller Aβ conformers *in vitro* (Muramatsu et al., 2025). Results after 78 weeks showed failure to achieve significance on either primary or secondary clinical outcomes in the overall study population (Abushakra et al., 2025). RHowever, there were small, but significant positive clinical effects in the MCI subset of participants. These findings await future direction based on the results of the ongoing Phase 3 clinical trial, introducing the idea that early-stage patients that are genetic carriers may be essential for devising precise therapeutics.

Blarcamesine is an orally available small-molecule activator of the sigma-1 receptor, a chaperone protein that resides at the endoplasmic reticulum and regulates autophagy (Malar et al., 2023). The rationale for use of blarcamesine in AD is that sigma-1 receptor activation may yield clinical improvement by restoring cellular homeostasis, stabilizing mitochondria and correcting autophagy dysregulation that is part of AD pathology (Christ et al., 2019; Drewes et al., 2025). In a double-blind, placebo-controlled, 48-week Phase IIb/III clinical trialNCT03790709, 508 participants aged 60–85 years with early AD were randomized to receive either capsules of blarcamesine 30 mg, blarcamesine 50 mg or placebo once daily (Macfarlane et al., 2025). The study found a significant increase in the plasma Aβ42/40-ratio in the blarcamesine treatment group vs. placebo as well as a significant decrease in loss of whole brain volume with treatment. In addition to demonstrating the safety and efficacy of blarcamesine, the clinical trial showed that blarcamesine significantly slowed clinical progression on prespecified primary outcome and cognitive measures at 48 weeks (Sabbagh et al., 2024). These results coupled with the convenient oral administration of the drug could suggest blarcamesine as a novel treatment option for early AD patients. Although some concerns have been expressed about the reporting of data on this drug, a Marketing Authorization Application for blarcamesine, has been submitted to the EMA while FDA approval has not yet been sought (Brodkin, 2025).

Targeting tau is an attractive modality for AD treatment because the scope of tau aggregation and spread is well-correlated with symptom severity in AD (Congdon et al., 2023; Van Alstyne et al., 2025). Older anti-tau therapies focused on post-translational modifications have been discontinued because of toxicity and lack of efficacy.

Small molecules with tau anti-aggregatory properties that have entered the clinical phase to date include methylene blue and low dose leucomethylthioninium bis-hydromethanesulfonate) (LMTM), a stabilized methylene blue derivative (Manglano-Artuñedo et al., 2024; Yang et al., 2024; Soeda et al., 2019). Methylene blue is a thiophenazine dye used in treating several illnesses including methemoglobinemia, septic shock, hepatopulmonary syndrome, and malaria (Gomez-Sequeda et al., 2024). It can cross the blood-brain barrier and had been molecule of interest, but there are no current clinical trials for this drug in AD (Hashmi et al., 2023). LMTM was studied in an 18-month phase III clinical trial NCT02245568in a non-randomized cohort of patients with AD (Thangwaritorn et al., 2024). The brain atrophy rate in patients on the monotherapy demonstrated a significant decline after 9 months of LMTM monotherapy (Wilcock et al., 2018). Another Phase 3 clinical trial with LMTM NCT03446001including 545 participants did not meet its co-primary clinical outcomes but did demonstrate a decline in brain atrophy (Wischik et al., 2022). A post-hoc analysis of a subgroup of patients on LMTM monotherapy with no other AD drugs exhibited a significant slowing in the rate of disease progression (15% of the total).

Neflamapimod, a small molecule oral drug inhibitor of p38 mitogen activated protein kinase α (MAPKα) is a small molecule with anti-inflammatory as well as neuro-regenerative effects (Jiang et al., 2022; Detka et al., 2024). The p38α MAPK pathway modulates synaptic plasticity, and when this pathway is disturbed under chronic inflammatory disease states, neurofibrillary tangle formation and synaptic dysfunction may ensue. Neflamapimod is currently in an active Phase 2a clinical study in patients with dementia and Lewy body formation which is in progress with 25 enrolled candidates (NCT06815965). Other studies have found a significant benefit on cognitive decline, but was conducted in persons with Lewy body dementia (Prins et al., 2024). Future studies evaluating the specific effect of neflamapimod on AD will give a more definitive picture of its efficacy in AD.

Another small molecule of interest is edaravone-dexborneol, which consists of edaravone and dexborneol at a 4:1 ratio, and is in clinical trials to test its efficiency in ischemic stroke treatment for its anti-inflammatory, antioxidants, and anti-apoptotic properties (Xu et al., 2021). It has recently been evaluated for possible application to AD. It reduces neuroinflammation in murine and cell culture AD models (Xu et al., 2021; Wang et al., 2024). Mao et al., 2025 showed that edaravone-dexborneoladminstrationis able to delay cognitive decline and synaptic loss in mice (Mao et al., 2025). These pre-clinical results have led to clinical studies for treatment of amyotrophic lateral sclerosis and to a Phase IIa clinical trial using oral edaravone, however results are not yet published (Yamashita and Abe, 2024; Oosthoek et al., 2023).

Lastly, mitochondrial dysfunction has been highlighted as a mechanism behind AD pathogenesis. Small molecules targeting mitochondria for AD therapy could be beneficial, although there is limited evidence in clinical trials (Pal, 2025). Acteoside is a phenylethanoid glycoside that has been reported to reduce Aβ and neurofibrillary tangle formation but also targets mitochondrial integrity and function by reducing ROS production (Zhang F.kun et al., 2025; Mohammed et al., 2025). However, the low bioavailability and unstable physiochemical properties of the drug limit its clinical application.

Newer tau-targeting agents are antisense oligonucleotides that function by binding and modifying RNA in a target-specific manner to slow disease progression (Frost et al., 2025; Lauffer et al., 2024). BIIB080, an antisense oligonucleotide that targets microtubule-associated protein, has been granted Fast Track designation by the US FDA, a process designed to facilitate the development and expedite the review of drugs intended to treat serious conditions, maintaining rigorous evidentiary standards while addressing unmet medical needs. An exploratory randomized placebo-controlled study of early AD patients receiving intrathecal BIIB080 over 13 weeks found that the treatment yielded a significant reduction in both total tau and phosphorylated tau 181 in CSF (Edwards et al., 2023). A systematic review of the impact of BIIB080 on tau biomarkers in patients with mild AD also found that it reduced total tau and phosphorylated tau 181 in CSF in higher-dose cohorts (Mahmoud and Ramadan, 2024). Positron emission tomography imaging demonstrated less buildup in 13 subjects compared to placebo by week 25. The Phase 2 study to determine efficacy and long-term safety is fully enrolled and data are expected in 2026.

While antisense oligonucleotides hold promise, the small molecules discussed here with various mechanisms have yet to prove clinical utility, and continued refinement of target selection and clinical integration is essential to overcome the complexity of AD pathology. In light of the multifactorial nature of AD, one or more of these options may prove useful in coordination with a regimen of therapies within a precision medicine approach to AD treatment.
